# Hepatic Inflammation and Liver Injury in a Model of Bacterial Infection Triggered Acute‐on‐Chronic Liver Injury

**DOI:** 10.1111/jgh.70055

**Published:** 2025-08-17

**Authors:** S. C. Karatayli, S. N. Weber, R. A. Hall, M. Hohl, L. Kaps, J. M. Schattenberg, F. Lammert, E. Karatayli

**Affiliations:** ^1^ Department of Medicine II Saarland University Medical Center Homburg Germany; ^2^ Department of Internal Medicine III Saarland University Hospital and Saarland University Homburg Germany; ^3^ Hannover Health Sciences Campus Hannover Medical School (MHH) Hannover Germany

## Abstract

**Background and Aim:**

Acute‐on‐chronic liver failure (ACLF) is characterized by acute decompensation of chronic liver disease in the presence of an acute trigger, and bacterial infection (BI) is the most common trigger of ACLF. Therefore, we aimed to establish a mouse model that mimics bacterial infection‐related acute‐on‐chronic liver failure (BI‐ACLF) to study the ongoing pathophysiological processes during disease progression.

**Methods:**

Wild‐type C57BL/6J (*n* = 12; wild‐type, WT) and *Abcb4* ^*−/−*^ (*n* = 12; knockout, KO) with underlying chronic fibrosing liver disease were intraperitoneally injected either with 0.9% NaCl or 4‐mg/kg lipopolysaccharide (LPS) to establish four experimental groups, namely, a control group (WT‐NaCl), an acute injury group (WT‐LPS), a chronic liver disease group (KO‐NaCl), and an acute‐on‐chronic group (KO‐LPS). Hepatic expressions (relative to *Gapdh*) of *Il‐6*, *Crp*, *Tnf‐α*, *Rantes*, *Tlr4*, *Mcp1*, *Il‐10*, *Il‐2*, *Il‐22*, *Il‐17a*, and *Tgf‐β* were quantified by the 2^−ΔΔCt^ method. Liver injury and inflammation were evaluated by Sirius red and H&E stainings, respectively. Immunohistochemical stainings were used to assess apoptosis (Ck‐18 and H2Ax), necrosis (Cas‐1 and Hmgb‐1), and macrophage polarization (M1 markers CD64 and CD86; M2 markers CD206 and Arg1). M1 markers (CD64 and CCR7) and M2 markers (CD163 and Arg1) were further analyzed by western blot analysis.

**Results:**

Hepatic cytokines and chemokines, monocyte chemoattractant protein‐1 (*Mcp‐1*), interleukins *Il‐2*, *Il‐22*, and regulated on activation, normal T‐cell expressed and secreted (*Rantes*) were significantly upregulated in mice of KO‐LPS groups compared to their counterparts. Induction of pyroptosis, apoptosis, and macrophage polarization towards the M1 phenotype was evident.

**Conclusion:**

Differential expression of hepatic cytokines and chemokines in *Abcb4* ^*−/−*^ mice upon LPS challenge provides insight into potential mediators of disease progression in this dual‐hit model of BI‐ACLI. Our findings suggest that increased expression of IL‐6, IL‐2, IL‐22, and RANTES may be associated with inflammatory responses that contribute to disease exacerbation in this refined model.

## Graphical abstract

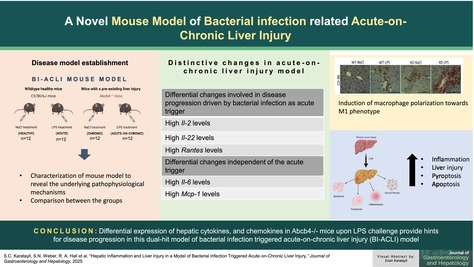

## 1 Introduction

Acute‐on‐chronic liver failure (ACLF) was first defined by a large prospective observational European study, named as “European Association for the Study of the Liver (EASL)–Chronic Liver Failure (CLIF) Consortium Acute‐on‐Chronic Liver Failure in Cirrhosis (CANONIC)” study [1], as a complex disorder of liver, characterized by the acute deterioration of pre‐existing chronic liver disease (CLD). The disease is usually associated with a precipitating event in the setting of advanced liver injury and cirrhosis. Bacterial infections (BIs) and active alcoholism are the most common precipitants of ACLF in this regard [2], resulting in a disease progression through acute decompensation (ad) of liver to hepatic or extrahepatic organ failures with high risk of short‐ and medium‐term mortality of 50%–90% [3, 4, 5]. The current treatment of ACLF mainly relies on management of the acute precipitating events and associated complications of the liver together with organ support providing. Besides, in cases of medical treatment failure, organ transplantation is left as the only treatment options for many patients with ACLF treatment. However, this is only available to a small subset of eligible subjects [6]. As revealed by the worldwide cohort studies, the clinical course of the disease is driven by three major stages, namely, the chronic liver injury, the acute precipitant, and the overreacting immune response particularly due to the following BIs causing systemic inflammatory response syndrome (SIRS) [1, 7, 8]. The driving force behind this imbalanced inflammatory response in ACLF patients is suggested to be mainly the acute precipitants in the settings of a chronically injured liver [9]. Despite accumulating studies on ACLF in recent years, the clinical pathomechanisms underlying the disease are still not well understood. Therefore, as reviewed elsewhere [10], different approaches have been used to establish animal models of ACLF to improve our understanding of the disease emergence and progression as well as to develop novel diagnostic, prognostic, and therapeutic options. With similar aims, we have previously established an alcohol‐related acute‐on‐chronic liver injury (ACLI) mouse model, in which National Institute on Alcohol Abuse and Alcoholism (NIAAA) chronic‐plus‐binge ethanol feeding model [11] was combined with the pre‐existing CLD in ATP Binding Cassette Subfamily B Member 4 knockout (*Abcb4* ^*−/−*^) mice, where an *Il‐6* driven acute inflammatory response was found to help transition from a stable chronic state to a progressive liver damage [12]. *Abcb4* ^*−/−*^ mice model has proven to be a useful tool to study the acute inflammatory responses in the background of a pre‐injured liver in this model. *Abcb4* ^*−/−*^ mice lack the ATP‐dependent phosphatidylcholine (PC) transport protein (multidrug resistance protein, MDR2), which flops PC across the canalicular membrane of hepatocytes. PC is an essential component of bile that forms vesicles to reduce the toxic detergent activity of bile acids. Therefore, the impaired biliary PC excretion in *Abcb4* ^*−/−*^ mice results in development of chronic progressive cholestatic liver injury, which is eventually followed by cirrhosis and hepatocellular carcinoma (HCC) at later ages [13]. In the present study, we establish and characterize a novel mouse model of ACLI in *Abcb4* ^*−/−*^ mice, using the BI as the acute trigger (BI‐ACLI).

## 2 Methods

### 2.1 Animal Studies

The *Abcb4* ^*−/−*^ mouse model used in this study has been well characterized in our previous publication, where chronic liver injury was confirmed by histological assessment of fibrosis and elevated alanine and aspartate transaminase (ALT/AST) levels in serum. *Abcb4* ^*−/−*^ mice develop progressive liver fibrosis characterized by bridging fibrosis by 15 weeks of age, as well as elevated serum markers of liver injury such as ALT and AST, establishing a robust CLD background suitable for studying ACLI mechanisms [12].

**FIGURE 1:**
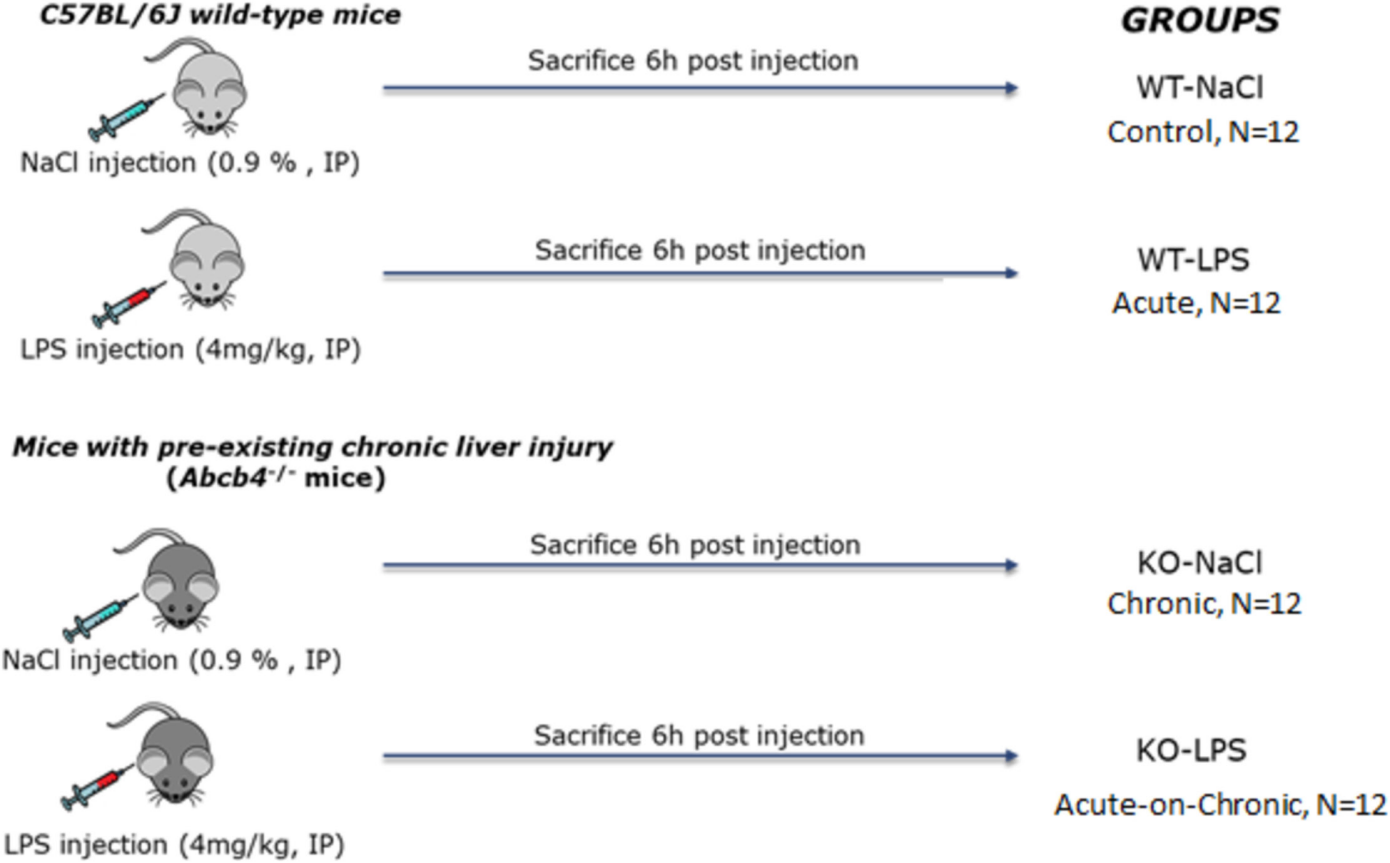
Graphical presentation of animal experiments. WT, wild‐type; KO, *Abcb4* ^*−/−*^; LPS, lipopolysaccharide; NaCl, 0.9% saline solution.

All mice were housed under environmentally controlled conditions (temperature, humidity 12/12‐h dark/light cycle) prior to saline buffer or LPS challenge. Administration of a 4‐mg/kg dose of LPS injection was based on a preliminary dose‐response study conducted in our laboratory (data not shown), as well as previously published studies demonstrating its efficacy in eliciting a robust inflammatory response [14, 15, 16]. Fifteen‐week‐old C57BL/6J wildtype mice and *Abcb4* ^*−/−*^ mice were then intraperitoneally injected with either a 0.9% NaCl solution or 4‐mg/kg LPS (from *Escherichia coli* Serotype 0111:B4 solution, Sigma) to establish WT‐NaCl (healthy control), WT‐LPS (acute liver injury), KO‐NaCl (chronic liver injury), and KO‐LPS (ACLI) groups (total *N* = 48; 12 for each group including 6 males and 6 females). Six‐hour postinjection mice were sacrificed (Figure 1). Plasma (from blood samples taken from inferior vena cava) and liver tissue samples were stored at −80°C for further use after freezing in liquid nitrogen. Relevant welfare regulations and the regulations of the Animal Care and Use Committee for Saarland University were applied in all animal study experimental protocols, which were approved by the Saarland University Animal Ethics Committee (TV26/2018).

### 2.2 Biochemical Analysis of Plasma

All laboratory data regarding biochemical analysis of the plasma, including the biochemical markers such as alanine aminotransferase ALT and aspartate aminotransferase AST, were carried out by the central laboratory of Saarland University Medical Center using the Roche cobas 8000 modular analyzer.

### 2.3 Characterization of Mouse Model; Liver Fibrosis and Inflammation

Assessment of liver fibrosis and inflammation was done by histochemical detection of collagen fibers by Picrosirius Red and hematoxylin–eosin (H&E) staining, respectively.

Liver samples were first fixed for 24 h in 4% neutral buffered formalin at 4°C and then embedded in paraffin. After staining of 3‐μm‐thick sections, histopathology was evaluated by a histomorphometric semi‐automatic system for image analysis (Leica microscope, equipped with Leica application suite software (LAS X) Wetzlar, Germany) as previously defined elsewhere [17]. Moreover, relative quantifications of *Col3A1* and *Crp* expressions in liver samples were also used to evaluate fibrosis and inflammation, respectively.

### 2.4 Relative Quantification of Hepatic Gene Expression Analyses

Total RNA extraction by RNeasy Mini Kit (Qiagen, Hilden, Germany) from snap‐frozen liver tissue samples was followed by the reverse transcription of 1 μg extracted RNA by using the High Capacity cDNA Reverse Transcription Kit (Life Technologies, Carlsbad, CA). Quantitative real‐time PCR was carried out in TaqMan 7500 Fast Real‐Time PCR System using the following predesigned TaqMan gene expression assays (Applied Biosystems/Thermo Scientific, Foster city, CA, USA): Interleukin 6 (*ll‐6*; Mm00446191_m1), C‐reactive protein (*Crp*; Mm00432680_g1), Collagen 3α1 (*Col3a1*; Mm01254476_m1), Regulated upon activation, normal T‐cell expressed and secreted (*Rantes/Ccl5*, Mm013002427_m1), Monocyte chemoattractant protein 1 (*Mcp1/Mcp‐1*, Mm00441242_m1), Toll‐like receptor 4 (*Tlr‐4*, Mm00445273_m1), Interleukin 10 (*ll‐10*, Mm01288386_m1), Interleukin 22 (*ll‐22*; Mm01226722_g1), Interleukin 2 (*ll‐2*, Mm00434256_m1), Interleukin‐1β (Il1b, Mm00434228‐m1), Tumor necrosis factor‐α (*Tnf‐α*, Mm00443258_m1), Transforming growth factor‐β1 (*Tgfb1*, Mm01178820_m1), Interleukin 17a (ll‐17a; Mm00439618_m1), and Glyceraldehyde‐3‐phosphate dehydrogenase (*Gapdh*, Mm99999915_g1). *Gapdh* was used as an internal control for relative quantification of the target genes by ΔΔCt method.

### 2.5 Western Blot

Mouse liver tissue was homogenized in homogenization buffer (5‐mmol/L EDTA; 25‐mmol/L NaF; 300‐mmol/L sucrose; 30‐mmol/L KH2PO4, pH = 7.0) containing mini complete protease inhibitors (#11873580001; Roche, Penzberg, Germany) and 1‐mmol/L PMSF. The homogenate was centrifuged at 13.000 rpm for 10 min, and the supernatant was transferred to a fresh incubation tube. Fifty micrograms of protein was separated on 10% SDS‐PAGE and electrophoretically transferred to nitrocellulose membranes (0.2‐μm pore size, #1620112, Bio‐Rad Laboratories Inc., Germany). Membranes were blocked in 1xPBS‐T (phosphate‐buffered saline: 137‐mmol/L NaCl; 2.7‐mmol/L KCl; 4.3‐mmol/L Na2HPO4; 1.47‐mmol/L KH2PO4, pH 7.4 containing 0.1% Tween) containing 5% nonfat dry milk for at least 120 min at room temperature and exposed to the following primary antibodies overnight: Cd64 (Abcam, ab203349), CD163 (Abcam, ab182422), CCR7 (Abcam, ab32527), and Arg1 (Abcam, ab233548). Respective secondary antibodies (purchased from Bio‐Rad Laboratories Inc., USA: anti‐mouse: #170‐6516, anti‐rabbit: #172‐1019) were incubated for 60 min at room temperature. Proteins were visualized by enhanced chemiluminescence according to the manufacturer's guidelines (#RPN2106, Amersham Pharmacia Biotech, Amersham, UK) and analyzed using the Fusion SL gel documentation system (Peqlab, Erlangen, Germany). Data are presented as integral optical density (IOD) normalized to GAPDH.

### 2.6 Immunohistochemistry (IHC)

IHC staining of paraffin‐embedded liver tissue sections (Hmgb1; Abcam ab18256, Arginase; Abcam ab96183, CD206; Abcam ab64693, CD68; Abcam ab125212, Ck18; Abcam ab315096, H2Ax; Abcam ab11175, CD64; Abcam ab203349, Cas‐1; Abcam Ab138483, CD86; Thermofisher Scientific PA5‐96112) was performed with 3‐μm‐thick paraffin slides, which were first deparaffinized in a descending alcohol series. Deparaffinization was followed by antigen retrieval in 10‐mM citrate buffer (pH 6.0) in a microwave oven (600 W, 15 min). Incubation for 20 min in 3% (v/v) H_2_O_2_ in PBS blocked the endogenous peroxidases. After blocking with an avidin and biotin system (Dako), slides were washed in PBS. The primary antibodies were diluted as 1:1000 dilutions in 2% (w/v) milk powder in PBS and then incubated overnight at 4°C (for antibodies Hmgb1, CD206, and CD86) or 1 h at 37°C (for antibodies Arginase, Ck‐18, H2Ax, CD68, and CD64). Biotinylated goat anti‐rabbit secondary antibody (1:100 diluted in 2% (w/v) milk powder in PBS) and ABC complex (Vectastain) were used after primary antibody incubation. Mayer's hematoxylin solution was used as the final step to counterstain the nuclei. In the case of H2Ax‐IHC, eosin, which stains cytoplasm, extracellular matrix, and collagenous connective tissue rather than nuclei, is used as the counterstain.

## 3 Results

### 3.1 Fibrosis and Inflammation

**FIGURE 2:**
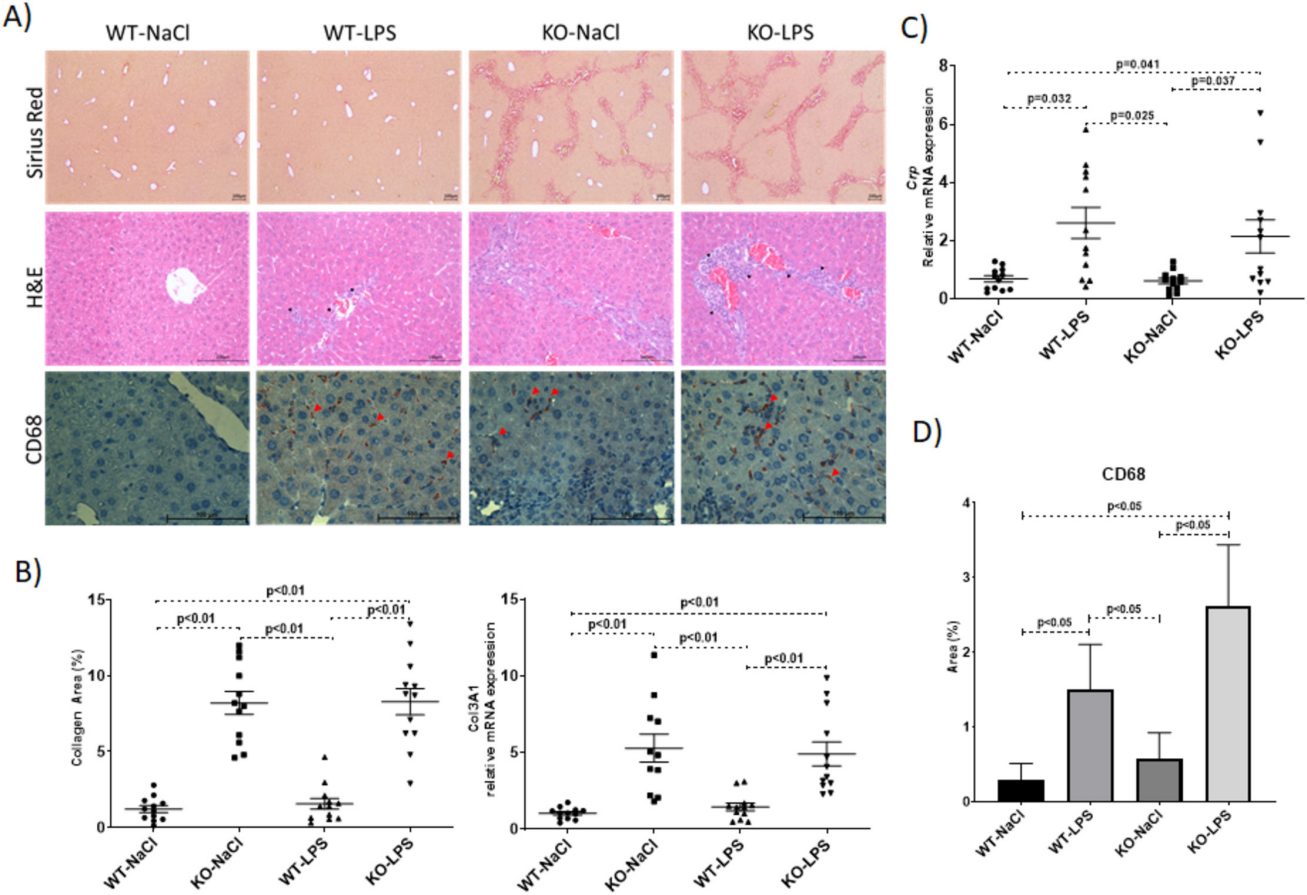
Evaluation of fibrosis and inflammation. Representative Sirius red‐stained and H&E stained liver sections and immunohistochemical staining of CD68 (A) semiquantitative analysis of hepatic collagen areas (%) and relative quantification of hepatic Col3a1 expression determined by qPCR (B) relative quantification of hepatic Crp expression determined by qPCR (C) and semiquantitative analysis of CD68 IHC staining. WT, wild‐type; KO, *Abcb4* ^*−/−*^; LPS, lipopolysaccharide, NaCl, 0.9% saline solution. CD68 positive stainings are shown by red triangles. WT, wild‐type; KO, *Abcb4* ^*−/−*^; LPS, lipopolysaccharide; NaCl, 0.9% saline solution. Scale bar, 100 μm.

Fibrosis stages ranging from none (in WT mice) to bridging fibrosis between portal areas (in KO mice) were observed, as shown by Sirius Red staining (Figure 2A, upper part). Significant collagen accumulation in *Abcb4* ^−/−^ mice was also confirmed by *Col3a1* steady‐state mRNA levels and collagen area (%) calculated from histopathological evaluation of liver sections performed using a semiquantitative scoring system (fibrosis scores) (Figure 2B). Significant inflammation was evident upon LPS injection, as demonstrated by increasing numbers of inflammatory infiltrates observed in H&E‐stained liver sections (Figure 2A, middle part) together with upregulation of hepatic *Crp* expression (Figure 2C). In accordance with these results, immunostaining of inflammatory marker CD68 (Figure 2A, lower part) has also revealed significantly higher levels of inflammation after LPS challenge (Figure 2D).

### 3.2 Plasma Surrogate Markers

**FIGURE 3:**
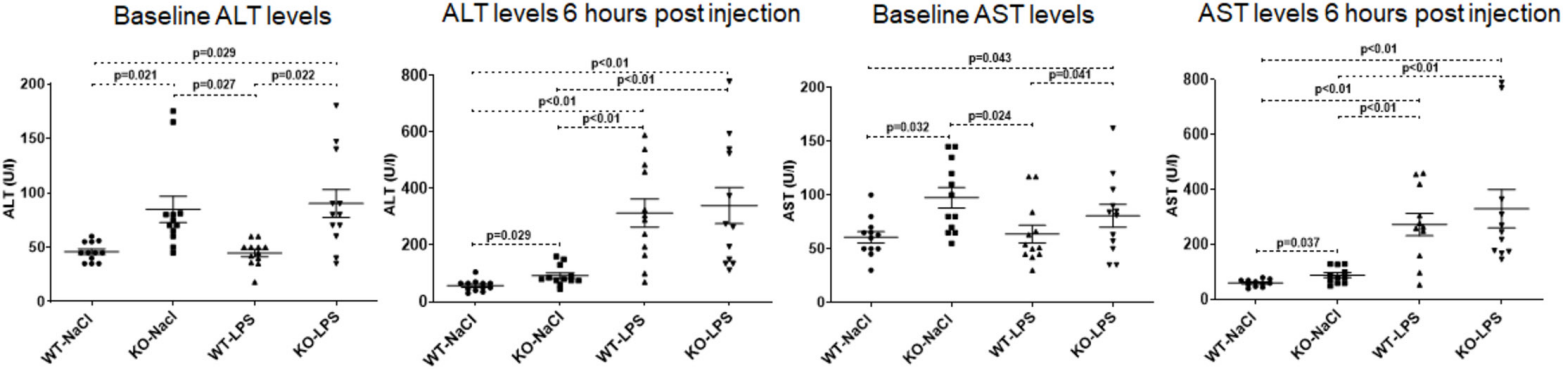
Biochemical markers. ALT and AST levels prior to LPS injection (baseline) and 6 h after LPS injection when mice were sacrificed. WT, wild‐type; KO, *Abcb4* ^*−/−*^; LPS, lipopolysaccharide; NaCl, 0.9% saline solution.

Baseline ALT and AST activities prior to LPS injection in plasma samples were significantly higher in knockout mice compared to controls (Figure 3). LPS injection resulted in a marked enzyme elevation both in wildtype and *Abcb4* ^−/−^ mice 6‐h postinjection.

### 3.3 Relative Hepatic Gene Expression of Inflammatory Mediators

**FIGURE 4:**
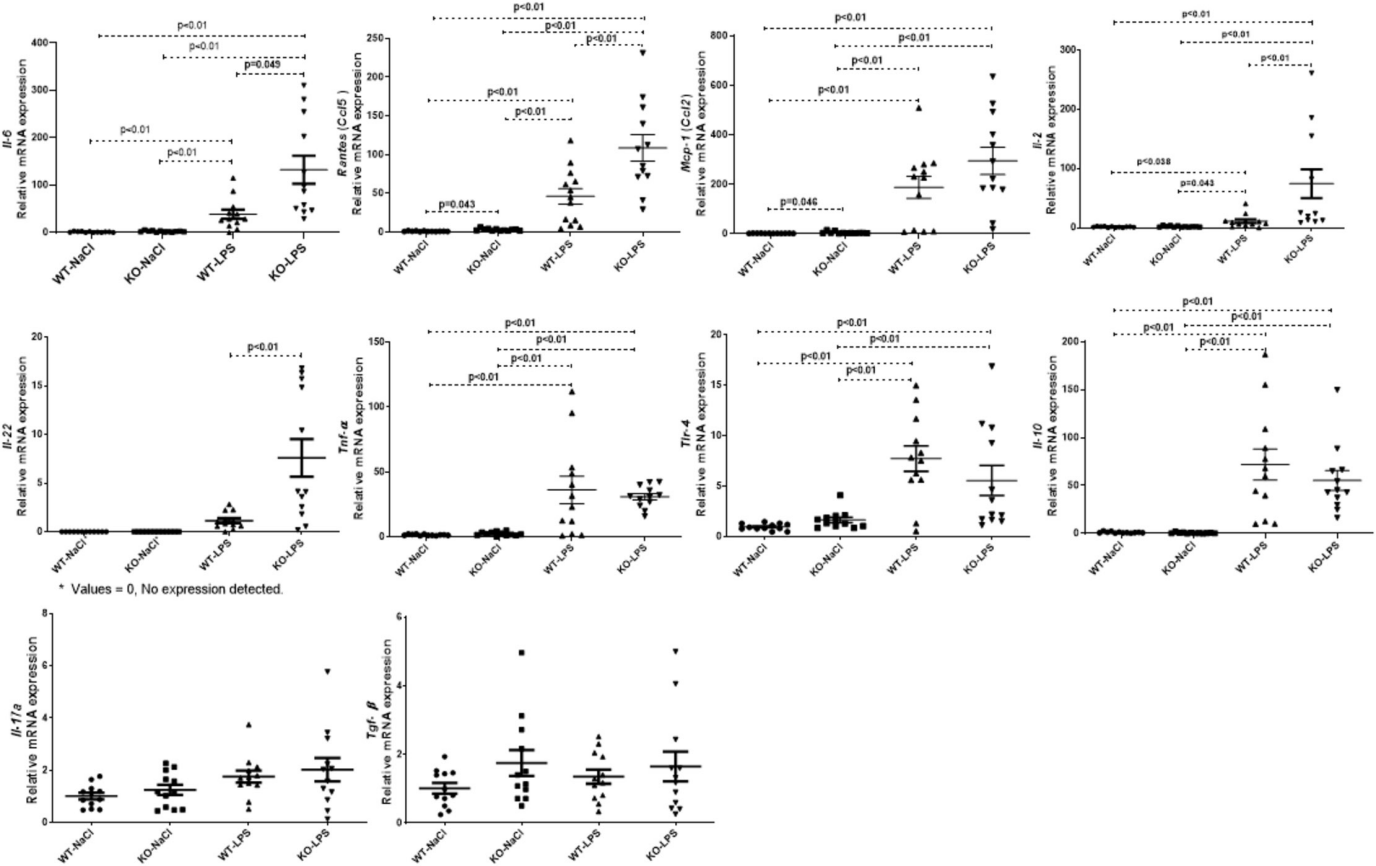
Relative hepatic gene expression of cytokines and chemokines, including Il‐6, Rantes, Mcp‐1, Il‐2, Il‐22, Tnf‐α, Tlr4, Mcp1, Il‐10, Il‐17a, and Tgf‐β. WT, wild‐type; KO, *Abcb4* ^*−/−*^; LPS, lipopolysaccharide; NaCl, 0.9% saline solution; Il, interleukine, Mcp‐1, Monocyte chemoattractant protein‐1; Rantes, regulated on activation, normal T‐cell expressed and secreted; Tnf, tumor necrosis factor; Tgf, transforming growth factor.

LPS challenge resulted in rapid upregulation of hepatic expression of toll‐like receptor 4 (*Tlr4*) (sixfold), while leading to a dramatic changes in *Mcp1*, *Il‐10*, *Rantes*, *Il‐6*, *Tnf‐α*, and *Il‐2* mRNA levels with approximately 200‐, 60‐, 40‐, 45‐, 30‐, and tenfold increases in WT mice, respectively (Figure 4). Similar corresponding effects were also observed in *Abcb4* ^*−/−*^ mice with no significant differences in terms of liver‐specific expression of *Tnf‐α*, *Tlr4*, and *Mcp1* as compared to wild‐type mice. Interestingly, LPS resulted in a more profound upregulation of *Rantes* (40‐fold vs. 120‐fold increase, *p* < 0.01) and *Il‐2* expression (fivefold vs. 75‐fold increase, *p* < 0.01) in *Abcb4* ^*−/−*^ mice with pre‐existing liver injury. A similar trend was also observed for *Il‐6* (45‐fold vs. 95‐fold increase, *p* = 0.053). Hepatic *Il‐22* mRNA expression was detected at baseline in neither wild‐type nor knockout mice, while LPS stimulated *Il‐22* expression in both genotypes with a sevenfold increase in knockout as compared to wild‐type mice (*p* < 0.01, Figure 4). On the other hand, *Tgf‐β* and *Il‐17a* levels did not differ in all groups, suggesting that neither the genetic background of the mice nor the LPS challenge had an effect on these cytokines.

### 3.4 Macrophage Polarization

**FIGURE 5:**
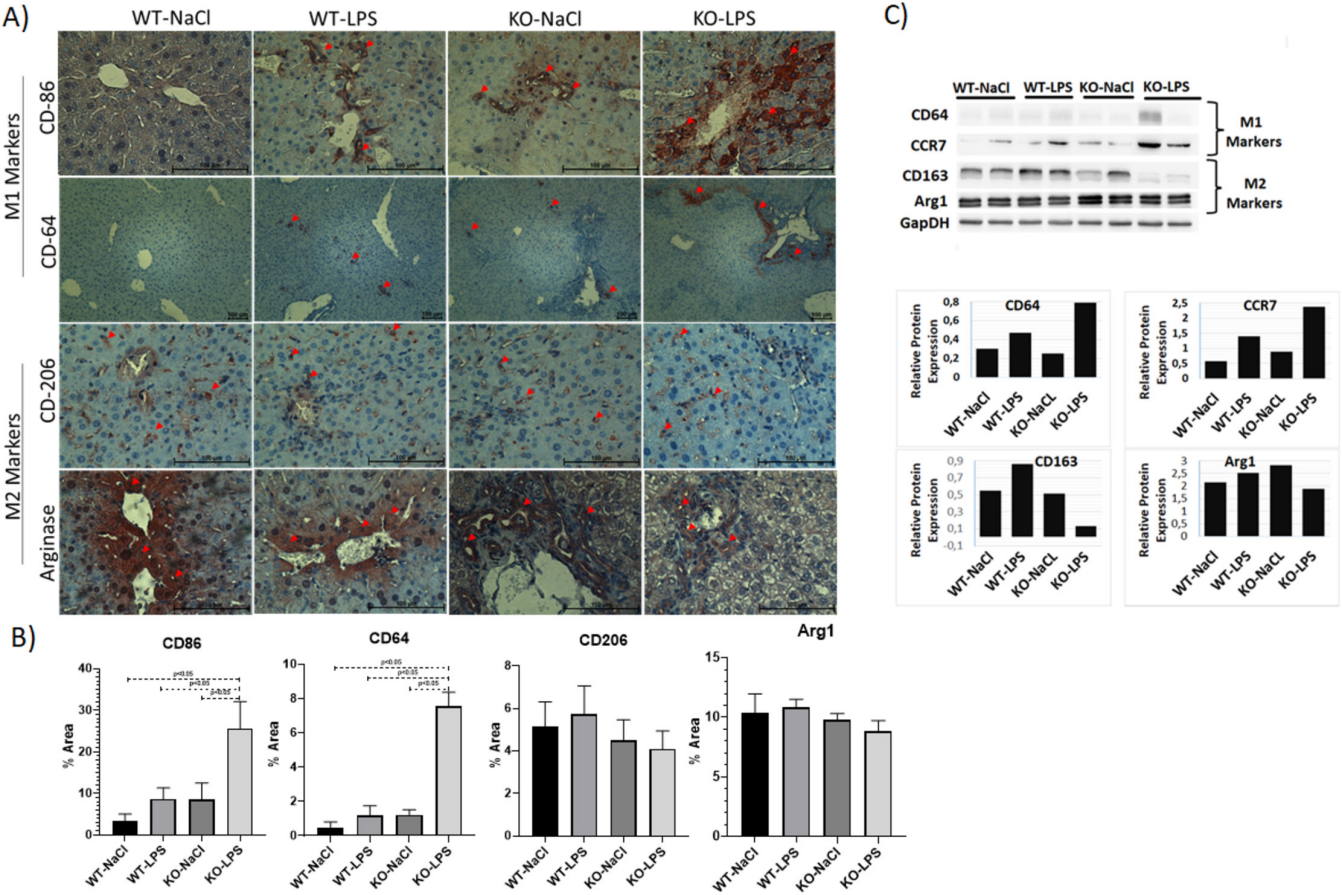
Macrophage polarization. Immunohistochemical stainings of M1 markers Cd64 and Cd86 and M2 markers arginase and Cd206 (A), semiquantitative analysis of IHC stainings (B), Western blot of M1 markers Cd64 and CCR7 and M2 markers Cd163 and Arginase and quantitation of relative protein expression of western blots (C). WT, wild‐type; KO, *Abcb4* ^*−/−*^; LPS, lipopolysaccharide; NaCl, 0.9% saline solution; CD, cluster of differentiation; Arg1, Arginase, CCR7, C‐C chemokine receptor 7. Red arrows show the positive ICH stainings. Scale bar 100 μm.

A macrophage polarization towards the M1 phenotype was evident by IHC stainings and western blot analysis of M1 and M2 macrophage markers (Figure 5A). M1 macrophage markers CD64 and CD86 have been significantly upregulated in the KO‐LPS group, while M2 macrophage markers Arginase and CD206 remained unchanged in IHC stainings (Figure 5A,B). Similarly, western blot analysis has shown that M1 macrophage markers CD64 and CCR7 have higher hepatic expressions in LPS‐challenged knockout mice, while hepatic expression of the M2 macrophage marker CD163 is downregulated and that of Arginase did not differ (Figure 5C).

### 3.5 Necrosis and Apoptosis

**FIGURE 6:**
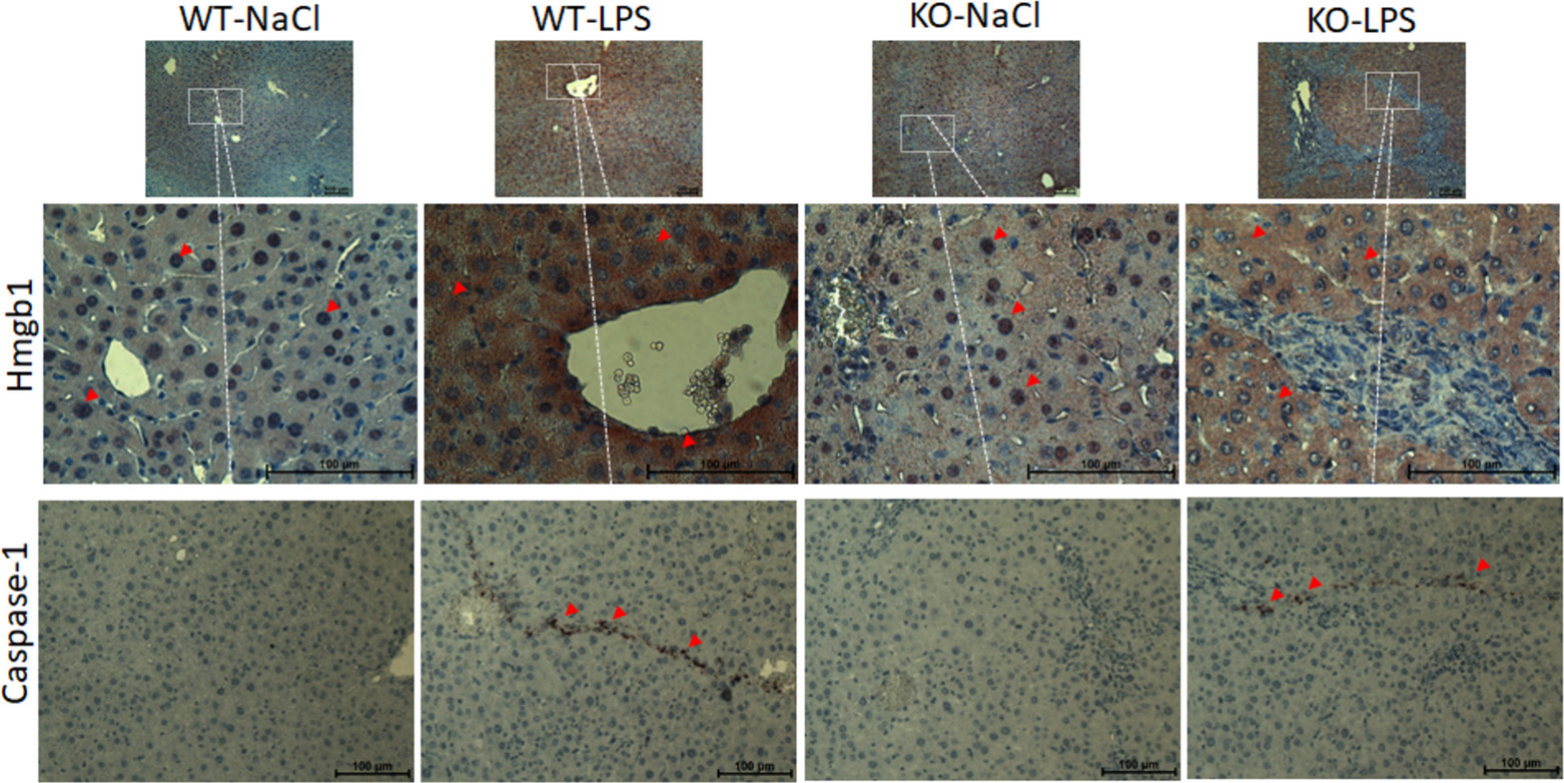
Evaluation of necrosis and pyroptosis. Immunohistochemical staining of Hmgb‐1 and caspase 1 cleavage. WT, wild‐type; KO, *Abcb4* ^*−/−*^; LPS, lipopolysaccharide; NaCl, 0.9% saline solution; Hmgb‐1, High mobility group box 1. Red arrows show the positive ICH stainings. Scale bar 100 μm.

**FIGURE 7:**
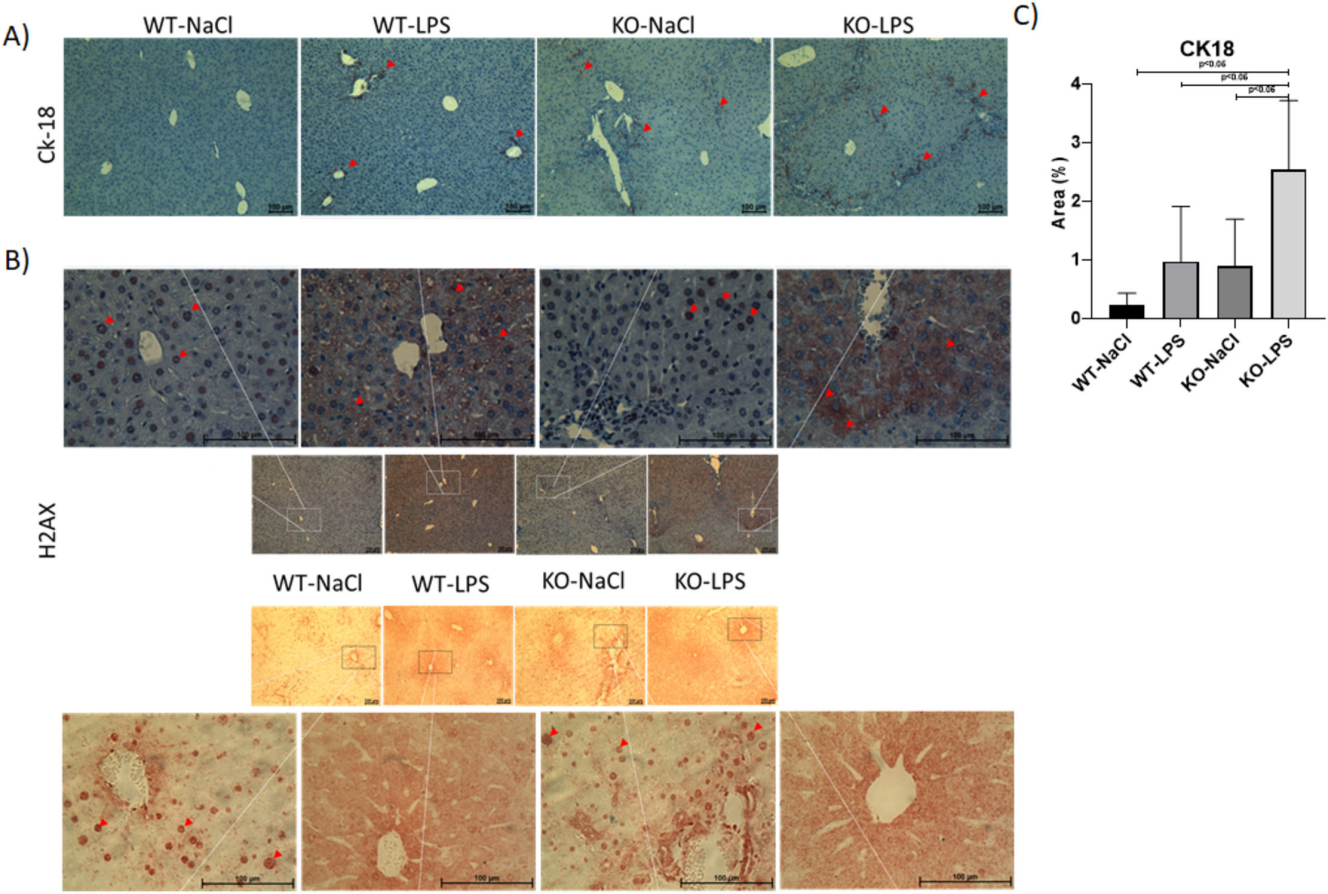
Evaluation of apoptosis. Immunohistochemically staining of apoptosis markers Ck‐18 (A) and H2Ax (B) and semiquantitative analysis of CK‐18 IHC (C). WT, wild‐type; KO, *Abcb4* ^*−/−*^; LPS, lipopolysaccharide; NaCl, 0.9% saline solution; Ck‐18, cytokeratin 18; H2Ax, H2A histone family member X. Red arrows show the positive ICH stainings. Scale bar 100 μm.

A distinct nuclear expression of necrosis marker Hmgb1 was evident in LPS‐nontreated WT and knockout mice by IHC staining (Figure 6). Diffusion of nuclear Hmgb1 to cytoplasm occurred upon LPS challenge as shown by dense cytoplasmic staining, which was accompanied by a positive IHC staining of pyroptosis marker Cas‐1. After observing a similar cytoplasmic diffusion of apoptosis marker H2Ax upon LPS challenge, eosin instead of hematoxylin was used as the counter staining in order for a better visualization of the cytoplasmic diffusion of H2Ax. Densely stained nuclei and cytoplasmic stainings of H2Ax were demonstrated in liver samples of LPS‐nontreated and LPS‐treated animals, respectively (Figure 7B). Moreover, quantification of IHC stainings of another apoptosis marker, Ck‐18, has shown that apoptosis is significantly upregulated in the KO‐LPS group compared to other groups (Figure 7C).

## 4 Discussion

Acute worsening of the previously well‐compensated CLD is the main feature of ACLF, which carries a high mortality. The onset of the acute transition from a compensated to decompensated stage of liver mainly relies on the precipitating factors. Currently, removal or treatment of the precipitants in addition to providing organ support and liver transplantation in eligible patients is the only treatment regimen in ACLF. In this context, BIs have been defined as the most frequent acute trigger of ACLF in various studies such as the European CANONIC study (40%) [1], a study with a Chinese cohort (%35) [18], the study of Fernandez and colleagues (37%) [19], and the study of Barosa and colleagues (40.7%) [20]. Of note, Mücke MM et al. reported a fourfold increased mortality when the precipitating event of ACLF is BI [21]. The aim of the current analysis was to establish a refined and simple model of ACLF and characterize it to allow for the development of novel treatment approaches. We have previously combined the chronic plus binge alcohol feeding model (NIAAA model) [11] with *Abcb4* ^*−/−*^ mice in order to study the inflammatory and immune responses in the alcohol‐triggered ACLI model [12], where highly significant overexpression of *Il‐6* and *Mcp‐1* in alcohol‐challenged *Abcb4* ^*−/−*^ mice highlighted the importance of the acute insult (alcohol) in pre‐injured liver (knockout mice). In the current study, ethanol challenge was replaced with LPS injection, which allowed us to compare the effects of different precipitants in these models. As reviewed by Dabrowska A et al., *Il‐6* is one of the main actors in the inflammatory response in ACLF [22]. Hence, it is not surprising that *Il‐6* overexpression was also present in our BI‐related ACLF model. Similarly, significantly high levels of MCP‐1 were detected in both models. In line with our models, many other animal models of ACLF [23] and several clinical studies [24, 25, 26, 27] evaluating the role of cytokines in ACLF with various precipitating events have shown increased levels of both *Il‐6* and *Mcp‐1* levels suggesting that these two inflammatory mediators may play a common role in ACLF disease progression independent of the acute trigger.

Unlike our alcohol‐related ACLI model, *Rantes*, *Il‐2*, and *Il‐22* were found to be significantly over‐expressed in the KO‐LPS group, emphasizing the distinct effect of BI on the pre‐injured liver. Among them, *Il‐22* has been shown to ameliorate ACLF in a mouse model established by CCl_4_ treatment in combination with *Klebsiella pneumonia* to mimic the BI [28]. The study has shown that the amelioration of ACLF was due to a shift in the hepatic IL‐22 signaling, which results in the reprogramming of impaired regenerative pathways through regulation of pro‐regenerative IL‐6/signal Transducer and Activator of Transcription 3 (IL‐6/STAT3) and anti‐regenerative interferon γ/signal Transducer and Activator of Transcription 1 (IFNϒ/STAT1) pathways. Moreover, administration of interleukin‐22 (Il‐22Fc) reversed the STAT1/STAT3 pathway imbalance, improving the survival of the ACLF mice. As reviewed by Hwang et al., Il‐22 has been suggested as a treatment option for various liver diseases, including ACLF, due to its well‐documented potency in protection against organ damage and reduction in BI in many animal models of liver disease and several clinical trials [29]. Il‐2 has long been known to be enhanced by LPS in vivo [30]. Besides, a more recent study has shown that cirrhosis‐associated immune dysfunction is driven by *Il‐2* [31]. Taken together, it is not surprising that *Il‐2* is significantly overexpressed in our model, where LPS is used as an acute challenge on chronically injured livers of *Abcb4* ^*−/−*^ mice. *Rantes*, together with *Mcp‐1*, has previously been defined as mediators of acute and chronic inflammation [32]. Moreover, it was shown to be released from LPS‐induced M1 polarized macrophages [33]. In various pathological conditions, including liver diseases, macrophage polarization plays significant roles. In a mouse model of acetaminophen (APAP)‐induced acute liver injury, loss of *Rantes* in *CCL5* ^*−/−*^ mice promoted M2 macrophage polarization [34]. The study has also shown that, by interacting with CCR1 and CCR5, *Rantes* promotes the M1 phenotype and inhibits the polarization to the M2 phenotype. Similarly, CCL5 expression was found to be highly correlated with M1 macrophages, but not with M2 macrophages in visceral white adipose tissue [35]. These findings may provide an explanation for the macrophage polarization towards the M1 phenotype in our model, since *Rantes* is highly significantly elevated in KO‐LPS group mice compared to their counterparts in other groups. Moreover, *Mcp‐1* was shown to recruit peripheral monocytes to the liver [36]; hence, its overexpression in our model also supports the transition to the M1 dominant phenotype in hepatic macrophages. The M1/M2 ratio usually correlates with the progression of many diseases, and our study has clearly shown that the dual hit (acute‐on‐chronic) in our model resulted in an M1/M2 phenotypic imbalance. Given that M2 macrophages are usually involved in resolution of inflammation, tissue repair, and regeneration [37], the observed imbalance in macrophage phenotypes might explain the disease progression in this ACLI model.

*Il‐17* and *Il‐22* have been well documented to play pivotal roles in host defense against infections [38]. Interestingly, although both have been reported to be produced predominantly from the same source, i.e., T helper 17 (Th17) cells, they were shown to be regulated differently in T cells [39]. Although *Il‐6* alone is sufficient to induce *Il‐22* production, the combination of *Il‐6* and *Tgf‐β* is required for *Il‐17* production. This distinct regulation was also evident in our model. We did not observe an upregulation of *Tgf‐β*, and hence *Il‐17* in our model, while *Il‐6* and *Il‐22* were significantly higher. Interestingly, *Il‐17* was also found to be involved in macrophage polarization and play an active role in promoting M2 macrophage differentiation [40], which further supports the observed macrophage differentiation in our model.

HMGB1 is a typical damage‐associated molecular pattern (DAMP), which can be translocated from the nucleus to the cytoplasm under various stress conditions [40]. As HMGB‐1 is reported as a necrosis marker [41], we evaluated the liver tissue samples IHC staining of HMGB‐1 to evaluate the necrosis. A previous study on sepsis, which defined HMGB‐1 as the endotoxin delivery protein, has shown that HMGB‐1 mediates the translocation of extracellular LPS to the cytosol leading to LPS interaction with caspase‐11 to trigger caspase‐11‐dependent pyroptosis, which is a highly inflammatory form of necrotic programmed cell death occurring mostly upon infection [42]. The same phenomenon was observed in our model as evidenced by HMGB‐1 IHC stainings, which showed the nucleocytoplasmic transport of Hmgb‐1 after LPS challenge and suggested ongoing cell deaths due to induction of pyroptosis in our model (Figure 6). Positive staining was subsequently observed with Cas‐1 IHC, a more specific marker for pyroptosis, following the detection of cytoplasmic localization of HMGB1 and H2AX. Similar cytoplasmic translocation was also detected for apoptotic marker H2Ax after LPS administration in our study (Figure 7B). Of note, the cytoplasmic localization of H2Ax was previously shown to be associated with tropomyosin‐related kinase A induced apoptosis [43]. Moreover, the quantification of IHC stainings of another apoptosis marker, Ck‐18, has revealed a significant upregulation in the knockout mice challenged with LPS suggesting that apoptosis, in addition to pyroptosis, is induced in the current model (Figure 7A).

Unfortunately, the pathophysiology of ACLF remains poorly understood. Animal models are very useful tools in this regard, which provide additional contributions to the accumulated data on disease pathomechanisms. However, there are some limitations of the current study in this regard. LPS administration does not fully replicate the complexity of real‐life BIs, which involve live pathogens and a broader range of immune interactions. Nonetheless, they remain valuable for investigating systemic inflammatory responses that contribute to liver inflammation, oxidative stress, and hepatocellular injury. These models effectively recapitulate critical features of sepsis‐induced liver injury observed in humans, thereby serving as relevant tools for studying acute liver dysfunction during sepsis [44, 45]. Secondly, while the observed changes in *Il‐6*, *Il‐2*, *Il‐22*, and *Rantes* expression suggest potential involvement in inflammatory processes, these findings are based on expression data alone. Additional functional experiments, such as gene knockout or inhibitor studies, would be beneficial to confirm the specific roles of these cytokines and strengthen the mechanistic conclusions.

In conclusion, the current study not only proposes a novel promising approach to model BI‐ACLI in *Abcb4* ^*−/−*^ mice with high expression levels of hepatic cytokines and chemokines after LPS challenge but also enabled us to compare the differences in ACLI disease progression arising from different precipitating events, namely, alcohol and BI, owing to the same background of chronic liver injury with our previous alcohol‐related ACLI model. Our data suggest that high differential expressions of *Il‐2*, *Il‐22*, and *Rantes* might be related to the inflammatory cascades specific to the BI‐ACLI model, while *Il‐6* and *Mcp‐1* driven inflammatory responses may represent a common pathway in the transition from a well‐compensated chronic state to a progressive liver injury independent of the acute trigger. Differential expression of these hepatic cytokines and chemokines resulted in induction of pyroptosis, apoptosis, and macrophage polarization in the current model.
